# Block-level vulnerability assessment reveals disproportionate impacts of natural hazards across the conterminous United States

**DOI:** 10.1038/s41467-023-39853-z

**Published:** 2023-07-14

**Authors:** Farnaz Yarveysi, Atieh Alipour, Hamed Moftakhari, Keighobad Jafarzadegan, Hamid Moradkhani

**Affiliations:** 1 Center for Complex Hydrosystems Research, University of Alabama, Tuscaloosa, AL 35487 USA; 2 Department of Civil, Construction and Environmental Engineering, University of Alabama, Tuscaloosa, AL 35487 USA

**Keywords:** Environmental social sciences, Natural hazards

## Abstract

The global increase in the frequency, intensity, and adverse impacts of natural hazards on societies and economies necessitates comprehensive vulnerability assessments at regional to national scales. Despite considerable research conducted on this subject, current vulnerability and risk assessments are implemented at relatively coarse resolution, and they are subject to significant uncertainty. Here, we develop a block-level Socio-Economic-Infrastructure Vulnerability (SEIV) index that helps characterize the spatial variation of vulnerability across the conterminous United States. The SEIV index provides vulnerability information at the block level, takes building count and the distance to emergency facilities into consideration in addition to common socioeconomic vulnerability measures and uses a machine-learning algorithm to calculate the relative weight of contributors to improve upon existing vulnerability indices in spatial resolution, comprehensiveness, and subjectivity reduction. Based on such fine resolution data of approximately 11 million blocks, we are able to analyze inequality within smaller political boundaries and find significant differences even between neighboring blocks.

## Abstract (web summary)

Introduces a precise, machine-learning-based Socio-Economic-Infrastructure Vulnerability index for natural hazards that uncovers stark variations in vulnerability at the block level emphasizing crucial information for risk-informed decision making.

## Introduction

The global increase in the number of recorded disasters caused by natural hazards necessitates the investigation of their adverse impacts and associated risks at the regional to local scale^1^. The term risk refers to the combination of hazard, exposure, and vulnerability^2^. All these variables are subject to various types of uncertainty, including those from input data, parameterization, and model structure^3,4^. Despite numerous studies on the spatiotemporal variability of hazards and exposure at local, regional, and global scales, the vulnerability aspect is still not well understood and has yet to be refined^5–11^. The term vulnerability refers to the circumstances shaped by physical, social, economic, and environmental factors or processes that heighten the susceptibility of an individual, a community, assets, or systems to the effects of hazards^12^. Such conditions/processes include but are not limited to predispositions, weaknesses, and lack of coping capacities favoring adverse effects of exposed elements, physical fragility of infrastructure, and blanket descriptors of social characteristics, i.e., age and gender^2^. Accurate quantification of all these components at a relevant spatial scale is key to successful risk assessment due to natural hazards^13^.

Current vulnerability assessment practices provide information at a resolution significantly larger than those that actual impacts are happening. Such a coarse resolution carries significant uncertainty in the estimates, and data at finer resolution have shown significant improvement in overall risk estimates^14,15^. Currently, the Centers for Disease Control and Prevention (CDC) provides the finest data on the spatial variability of the social vulnerability index (SVI) at the census tract scale over the Conterminous United States (CONUS)^16^. SVI, however finer than other alternatives, helps assess vulnerability at the scale of cities, towns, or other administrative areas. This is despite the fact that different neighborhoods or block groups within a city, based on their different socioeconomic characteristics, could demonstrate different levels of vulnerability to natural hazards^17^.

The compilation of data on qualitative and quantitative risk factors and subjectivity in the process of determining the weight/contribution of each factor to the overall risk has remained challenging. For instance, for the SVI, to calculate the overall index for each census tract over the CONUS, the contribution of all variables has been considered to be equal^18^. Schmidtlein et al.^19^ used principal components analysis (PCA) to examine the sensitivity of quantitative features underlying the social vulnerability index (SoVI) developed by Cutter et al.^20^. Additionally, to improve the socioeconomic vulnerability index (SEVI), Khajehei et al.^14^ and Tanim et al.^13^ applied probabilistic PCA (PPCA), which copes with missing values in the data.

Here, we develop a Socio-Economic-Infrastructure Vulnerability (SEIV) index that helps characterize the spatial variation in vulnerability across the CONUS at the census block level. The SEIV index benefits from already existing social, economic, and infrastructure indicators available via federally supported databases to improve upon vulnerability indices currently used for natural hazards risk assessment. To conduct a more comprehensive assessment and take the accessibility of critical infrastructure during hazardous situations into account, in addition to the commonly used demographic and housing information, for each block, we consider the building count and its distance to the nearest emergency/essential facilities. Using the variance inflation factor (VIF), we detect multicollinear variables and remove them from the dataset to avoid redundant information and alleviate the complexity of the input data. One significant contribution of the proposed SEIV in the process of risk assessment is reducing subjectivity in determining the contribution of various social, economic, and infrastructural components to overall estimated vulnerability. For this purpose, we use a machine learning (ML) algorithm to objectively assign weights to variables contributing to overall estimated vulnerability based on the significance of the contribution to the reported damage in the past. Such an objective weighting scheme helps ensure that the generated results are globally acceptable so the developed tools and techniques are transferrable to other places.

## Results

### Block-level vulnerability distribution

**Fig. 1:**
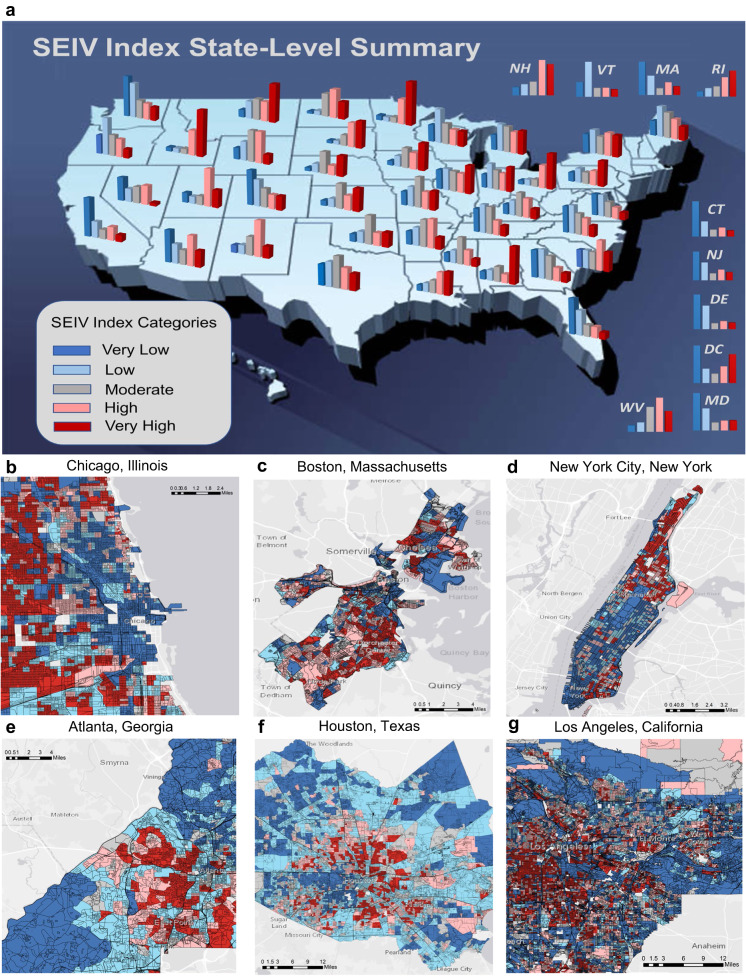
Spatial distribution of Socio-Economic-Infrastructure Vulnerability (SEIV) index statistics over the conterminous United States (CONUS). The upper panel (**a**) depicts the distribution of SEIV at an aggregate level (here, state level). The lower subpanels (**b**–**g**) represent the block-level distribution of SEIV in six large metros of the United States. NH New Hampshire, VT Vermont, MA Massachusetts, RI Rhode Island, CT Connecticut, NJ New Jersey, DE Delaware, DC District of Columbia, MD Maryland, WV West Virginia.

Current aggregate-level vulnerability indices favor top-down risk mitigation approaches and fail to engage local communities in improving resilience. Demographics and infrastructural characteristics can be drastically different between two neighboring blocks. This means indices that aggregate these features at larger scales (i.e., county level) may not resolve the spatial distribution of vulnerability characteristics well and thus pose significant uncertainty to risk estimates. Decisions made based on such information are not subsequently well-informed and thus are subject to failure and eventually yield to distrust in government. Different indices are currently in use for operational risk management, for example, integrate underlying featuring characteristics over the county of Harris or City of Houston^16^ to provide one single vulnerability estimate over the entire unit of interest (county or census tract). Such indices simply fail to locate communities/neighborhoods with an immediate need to pay attention to implementing risk mitigation measures and implement bottom-up approaches for adaptation. In fact, with such aggregate level assessment, there is no way to determine the relative urgency of action and resource allocation for improved resiliency. This yields in one-fit-all top-down governance that simply ignores existing variability in vulnerability dynamics at the community level, which in turn yields an increased likelihood of failure^21^. An index that can resolve heterogeneity of vulnerability to natural hazards at finer scales, i.e., block level, as does by SEIV, plays a key role in effective risk management and has the potential to provide crucial information for stakeholders at different levels for enhanced risk-informed decision making. Figure 1 presents the summary statistics of the percentage of blocks within each state that fall under various vulnerability categories defined by SEIV, along with the spatial distribution of vulnerability for six of the largest metros in the United States. Moreover, such information, when publicly available, will facilitate communications between local community leaders, non-governmental organizations (NGOs), and local/federal governments to build trust, synergize resources over agreed-upon strategies and mobilize resources to increase resiliency against upcoming hazards.

**Fig. 2:**
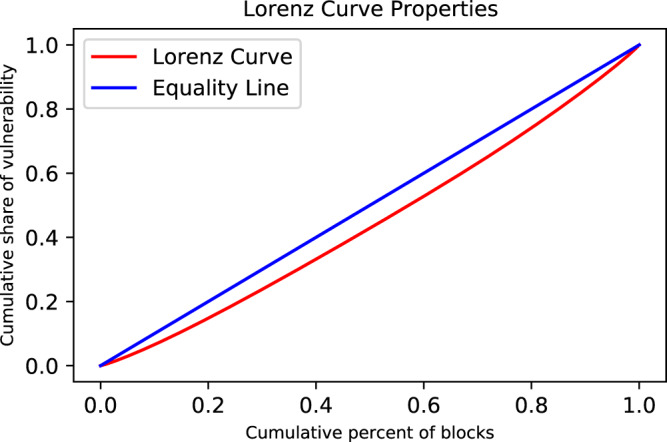
Lorenz curve properties for the SEIV (socio-economic-infrastructure vulnerability) index distribution over the CONUS (conterminous United States). The Lorenz curve and equality line are shown in red and blue, respectively.

Communities across the CONUS are not equally susceptible to upcoming hazards. There exists an inequality in vulnerability at various scales (i.e., between blocks, counties, and states). Figure 2 shows the Lorenz curve^22^ properties of SEIV index inequality over the CONUS. The Gini coefficient is a measure of inequality within a social group and is calculated based on the difference between the Lorenz curve (the observed cumulative distribution) and the notion of a perfectly equal distribution. The coefficient ranges from 0 to 1, where 0 represents perfect equality, and 1 indicates perfect inequality. The vulnerability Gini coefficient here is estimated to be 0.11, which demonstrates that communities across the CONUS will be disproportionately affected by natural hazards. While the 20% least vulnerable blocks hold a 15% share of vulnerability, the 20% most vulnerable hold 25% of the cumulative share of vulnerability.

Among the states all over the CONUS, Idaho (84%), Minnesota (82%), Ohio (76%), South Dakota (74%), New Hampshire (0.67%), Utah (67%), Rhode Island (66%), Louisiana (61%), North Dakota (0.60%), West Virginia (0.58%), and Alabama (57%) are the top 10 states with the highest percentage of blocks with high and very high SEIV indices. This is despite the fact that Ohio and Minnesota are among the top 10 states in GDP (gross domestic product) ranking and have relatively good standing in income Gini ranking^23,24^. This demonstrates how aggregate-level indices can misguide the process of vulnerability assessment and further highlights the need for an index that provides reliable vulnerability information at the finest resolution. On the other hand, Delaware (0.75%), Maryland (69%), Florida (68%), Vermont (67%), Massachusetts (0.66%), California (0.65%), Connecticut (0.65%), Washington (0.61%), New Jersey (0.59%), and Colorado (0.57%) are the top 10 states showing low and very low SEIV indices.

### Contribution of various components to the overall vulnerability

**Fig. 3:**
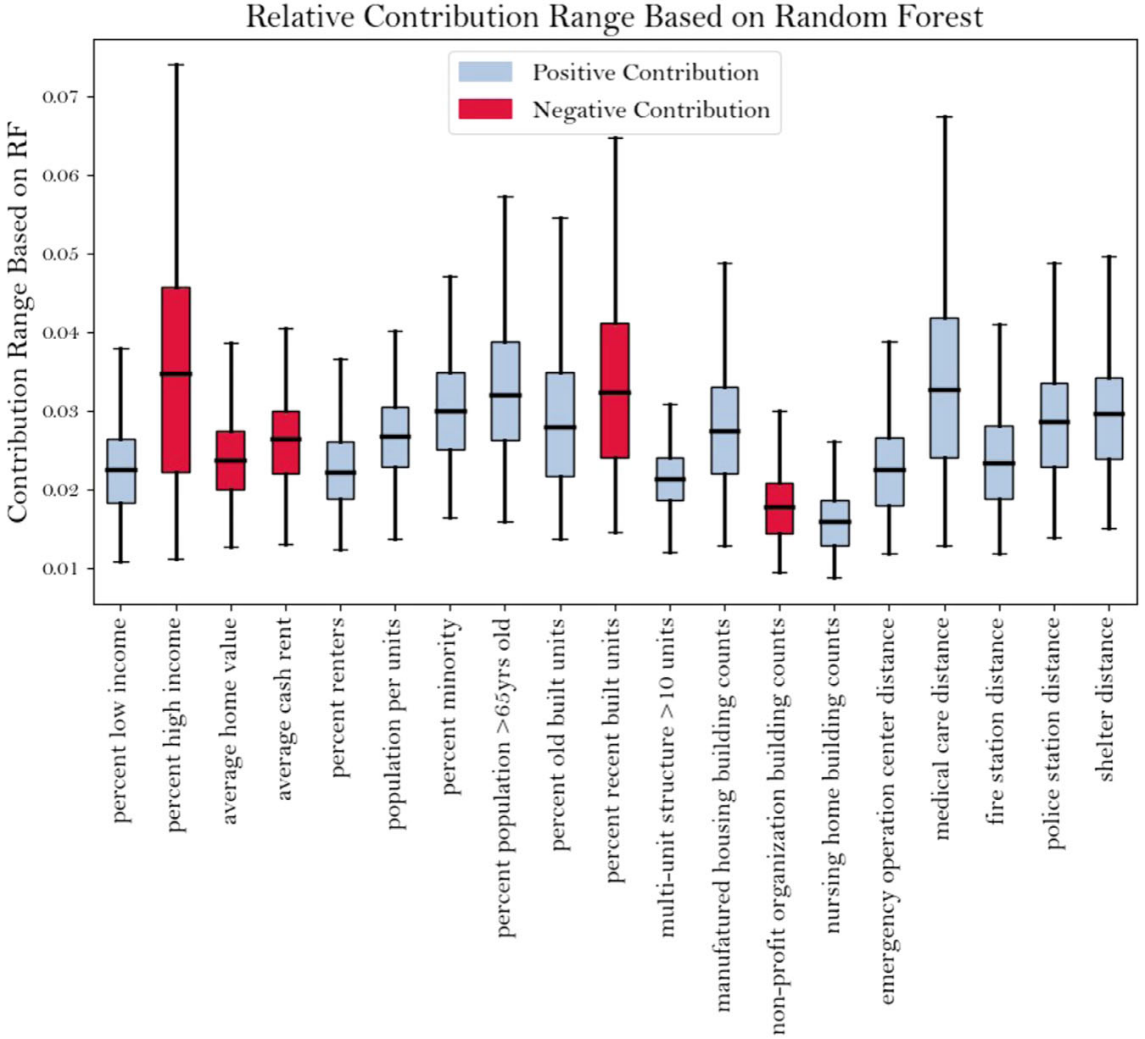
Contribution range of various factors in Socio-Economic-Infrastructure Vulnerability (SEIV) index based on random forest (RF) regression. Blue (red) represents a positive (negative) contribution to SEIV. The bounds of boxes are 25th and 75th percentiles, while the whiskers represent the minima and maxima, respectively. These box plot statistics are derived based on *n* = 130,481 independent events.

Vulnerability components do not equally contribute to the adverse impacts of natural hazards. Here, we waived the conventional assumption of the equal contribution of underlying components to overall vulnerability (e.g., via the rank percentile method in SVI). Our analysis, based on reported property damage costs, uses an ML algorithm to assign weights to various variables that contribute to vulnerability. The proposed model performs well in estimating the damage from validation events, which are not used in training. The results based on our model show a difference in the relative contribution of the components involved (Fig. 3). The *medical care distance, persons 65 years and older, distance to a shelter, percent minority* (e.g., various ethnicities and races), *and percent old built units* are found to be the most correlated descriptors of vulnerability (i.e., the larger these are, the higher the vulnerability is). On the contrary, *the percent high income, percent recently built units, and average cash rent* are the best counter-related predictors of vulnerability. The weights obtained from this approach help reduce subjectivity in determining the relative contribution of vulnerability factors and thus help provide a more realistic depiction of vulnerability distribution across the CONUS than what was available before.

It should be noted that the currently detected patterns of contribution for different components (i.e., increasing/decreasing with the vulnerability of an area) are conditioned on the pre-filtering practice implemented in this study that disregards relatively very high and very low-cost events in the process of training. This means that, in this analysis, given the fact that the events with more than $250 M reported damage are excluded from the training population, higher income/housing costs and lower incomes/housing costs have a negative and positive contribution, respectively, to property damage from storm events; which is in contrary to similar research in this topical area (i.e., Bolin and Stanford 1998^25^; Tierney 2006^26^; Osberghaus 2021^27^). Hence, our results are valid over the range of medium common possibilities of hazard events at which high-income households with sufficient resources and flexible planning capacities are less vulnerable to hazard situations and able to cope with and recover from them much easier than low-income families. Such difference between ours and similar studies in the recognized patterns stems from the nonlinear relationship between income/housing cost and property damage over different ranges of event costs.

Various factors might have contributed to the current distribution of vulnerability. Lack of both physical (e.g., transportation) and nonphysical infrastructure (e.g., insurance) makes low-income families dwell in hazard-prone areas, and their limited recovery capacity exacerbates the situation over time, especially when major hazards hit the area^28^. On the other hand, high-income households with sufficient resources and flexible planning capacities avoid such losses over time, so the inequality of vulnerability deepens over time, especially after major disasters. Infrastructural characteristics play a crucial role in resilience management^29^. In fact, vulnerability depends on the capacity of communities to cope with and recover from hazards. Without appropriate characterization of these aspects, i.e., accessibility of critical facilities during and after hazards, vulnerability assessment solely based on social and economic indices may mischaracterize the vulnerability. SEIV improves upon the antecedent indices by integrating information about various categories of building counts (e.g., multidwellings, manufactured housing, nursing homes, and nonprofits) and distance to essential facilities (i.e., emergency operation centers, shelters, medical care, fire stations, and police stations). This extra layer of information is a significant step towards a more comprehensive vulnerability assessment, which was not possible when aggregated at larger levels. In fact, the block-level assessment of vulnerability enables such detailed analysis of infrastructural heterogeneity and thus significantly increases the reliability of the assessment results for decision-making. SEIV, which takes infrastructural characteristics into account, helps reduce this gap via communicable assessment at the scale relevant to bottom-up resilience initiatives.

## Discussions

The proposed SEIV index is a valid vulnerability index that meets the widely accepted criteria, including practicality, transparency, interpretability, relevance, theoretical consistency, internal consistency, and external consistency^30^. SEIV is practical and transparent as the data and method are both publicly available, and so the results are reproducible and usable by interested scientists and practitioners. The range of variability (between 0 and 1) for SEIV makes it interpretable. The closer to zero, the lesser vulnerable the community is, and as the number grows and gets closer to one, vulnerability increases. Such a straightforward indexing system provides interpretable information and so makes the process of risk assessment and communication easy among potential end users. Relevance is ensured in SEIV index development through an expert opinion-based input variable selection procedure. The SEIV index benefits from already existing social, economic, and infrastructure indicators available via federally supported databases to improve upon vulnerability indices currently used for natural hazards risk assessment. To conduct a more comprehensive assessment and take the accessibility of critical infrastructure during hazardous situations into account, in addition to the commonly used demographic and housing information, for each block, we considered building count and its distance to the nearest emergency/essential facilities. Therefore, considering the concept of vulnerability assessment, all variables used in the SEIV index play a major role in quantifying the susceptibility of an individual, a community, and/or their assets to the impacts of hazards. SEIV is theoretically consistent, as it is estimated based on the classic theory of risk that characterizes risk as the combined effects of hazard and vulnerability. While there exists no adequate criterion/measure available to explicitly quantify the vulnerability, one can reasonably use proxies (i.e., reported damage) to characterize the risk and so validate the proposed measure of vulnerability. Moreover, here we use Random Forest (RF) algorithm to determine the contribution of the selected variables to the SEIV index in terms of built-in feature importance weights. It is an algorithm widely used in various fields of science (e.g., Biau and Scornet, 2016; Abbaszadeh et al., 2019)^31,32^. To check the internal consistency and robustness criteria, we use the K-fold cross-validation method to estimate the skill of the model on unseen data. To evaluate the performance of the algorithm, we used K-fold cross-validation (with *K* = 10). This approach helps stabilize the variability of accuracy estimates^33^. The performance metrics in estimating the damage from validation events, which are not used in training, can help appropriately evaluate the external consistency of the proposed indexing system. However, as mentioned earlier, in the absence of an explicit quantification for vulnerability, a thorough analysis of this type of consistency is not straightforward and must be done through proxy measures.

There are challenges in keeping the SEIV index up to date. First, census blocks are defined based on visible/invisible features upon adding/losing urban features due to urbanization or in response to natural disasters. Such changes and their associated data updates (via Hazus, for example) are released within 3–4 years after each decadal census revision^34^. The inconsistency of such data updates and changes made by anthropogenic or natural processes pose a challenge in keeping the SEIV database up to date.

Incompleteness and/or incomprehensiveness of data at the block level is another challenge to tackle for the most accurate SEIV indexing system. For a more comprehensive SEIV index, access to important aspects of vulnerability information, such as disability, education, unemployment, or language status, which are not currently available at the block level, would be very helpful. Additionally, for a more accurate training of ML algorithms, in addition to latitude and longitude, access to storm event damage data that provides information regarding the spatial extent to which damage has been estimated would be helpful.

Also, the inherent noise in the Hazus data that stems from the nature of block-level resolution at the census is another consideration of this index. Census block demographic data has notable noises infused to protect privacy; this would propose some uncertainty in the estimated SEIV index using block-level data in this study. Although it has not been done yet, if possible, denoising the publicly available data or accessing the raw data for an in-depth analysis of variability in various factors contributing to socio-economic and infrastructure vulnerability would help overcome this issue. Moreover, it should be noted that block-level Hazus data is based on longitudinal employer-household dynamics with a set of known nonsampling errors, including misreported data, late reporters whose records are missing and imputed, and geographic/industry edits and imputations. Where fine-resolution data with good accuracy is available, a thorough uncertainty analysis to determine the contribution of each of these potential sources of error would help make better estimates of vulnerability.

While our aim here is to assess relative Social, Economic, and Infrastructural vulnerability for all blocks over the entire CONUS and propose a single weighting system, some forms of classification might be considered helpful in the follow-up studies. For example, some components that show a stronger positive correlation with vulnerability (e.g., manufactured housing counts, distance to emergency facilities, and percent of the population over 65) are characteristics more common in rural areas, while some others that happen to show a negative correlation with vulnerability are characteristics typically associated with urban/metropolitan areas (e.g., average home value, average cash rent, percent high income). Thus, the stratification of results by urban-rural classification might provide valuable context for interpretation. However, the subjectivity of such stratification (urban vs. rural) at a large number of blocks over the CONUS would pose significant uncertainty to the calculated index. Considering urban/rural characteristics of a block needs complete and comprehensive data with an agreed demarcation to determine whether any block over the CONUS belongs to an urban or rural area.

## Methods

Providing the spatial distribution of block-level vulnerability across the CONUS is the main objective of this study. The descriptive social and economic characteristics data at the census block level, the smallest geographic area for which the Bureau of the Census collects and tabulates decennial census data, are available via U.S. Census Bureau every 10 years^34,35^. Blocks are formed by visible features such as streets, roads, railroads, streams, and other visible physical and cultural features and nonvisible boundaries such as property lines, cities, school districts, and county limits shown on Census Bureau maps^35^. We obtained descriptive components (demographics and building count by census block) from the Hazus data inventory through the Comprehensive Data Management System (CDMS). Hazus, a natural hazard modeling application developed by the Federal Emergency Management Agency (FEMA), provides information on potential loss estimates from floods, hurricanes, earthquakes, and tsunamis. CDMS is a complementary tool used to search for and transfer data into and out of a given Hazus state inventory dataset. The data available in Hazus 5.0, released on April 30, 2021, contain 59 and 33 demographic and building count variables, respectively, for 10,946,768 blocks over the CONUS. It is based on 2010 census data and modified by the National Structure Inventory (NSI) data developed by the United States Army Corp of Engineers Hydrologic Engineering Center, Flood Impact Assessment (USACE HEC-FIA). This modification, in coordination with FEMA, has been based on land cover patterns to include areas where structures are most likely to be found and to remove undeveloped areas such as areas covered by bodies of water, wetlands, or forests to avoid overestimation of losses. Additionally, the General Building Stuck (GBS) data in Hazus that is used here are based on RSMeans (a construction cost estimators toolbox) version 2018^34^.

One distinguishing aspect of SEIV is the consideration of the distance from each block to the nearest emergency facilities. For this purpose, the geographic information of emergency facilities, including fire stations, medical care facilities, shelters, and emergency operation centers, is obtained via Hazus, which was updated by the 2019 Homeland Infrastructure Foundation-Level Data (HIFLD)^34^. We have also used the HIFLD database to obtain geographic information on police stations (local law enforcement locations) and national shelter system facilities that have been assigned as shelters by either FEMA or the American Red Cross. The geographic information of blocks (i.e., latitude and longitude) for distance calculations are available via Census’s Block-level Geographic Information^36^. It should be noted that, if it was available, the inclusion of transportation infrastructure and access modeling could have improved the outcome of this analysis as compared to the currently implemented direct distance measure. While its development is beyond the scope of this work, such a model should also consider that some disastrous situations may interrupt the serviceability of transportation infrastructure, and an ideal model needs to take these factors into account for an appropriate accessibility assessment during hazardous situations.

To train and validate the developed ML algorithm, we use the National Oceanic and Atmospheric Administration (NOAA) Storm Events database, a comprehensive repository that provides information for different types of natural disasters between 2006 and 2020^37^. We used the database to obtain the location and property damage for 130,481 events with estimated damage between $10,000 and $250 million to ensure that the regression analysis results remained valid over the range of common possibilities. Exclusion of relatively very high (>$250) and very low (<$10,000) cost incidents from the damage data helps prevent the effect of hazard intensity overshadows the contribution of vulnerability. In fact, a relatively high level of damage (i.e., greater than $250) requires large-scale weather events under which a detailed analysis of the contribution of vulnerability components to the resulting risk is not possible^25^.

**Fig. 4:**
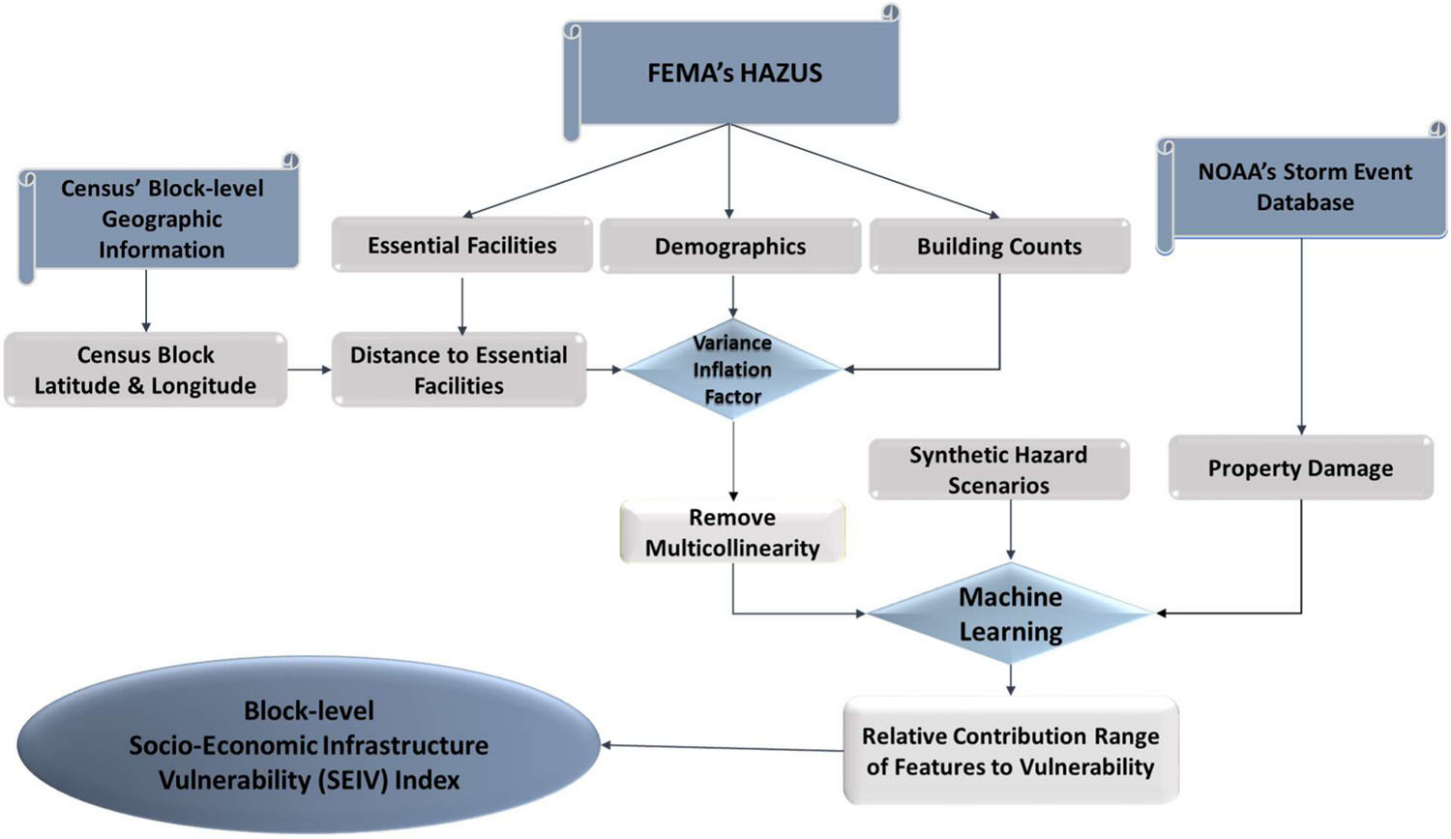
Flowchart of estimating the SEIV (socio-economic-infrastructure vulnerability) index. FEMA’s HAZUS refers to the natural hazard modeling application developed by Federal Emergency Management Agency, and NOAA is the National Oceanic and Atmospheric Administration. The machine learning algorithm used here is the Random Forest.

We developed a comprehensive block-level SEIV index that would be useful for high-resolution hydroclimatic risk assessment over the CONUS. Figure 4 demonstrates the overall flow of tasks/processes to calculate SEIV. To make information consistent and reliable for analysis, we first need to sort the data obtained from various sources and select the necessary variables for this study. Next, among 91 variables describing various aspects of socioeconomic vulnerability available via Hazus, we select the 38 most relevant components based on the existing literature^14,16,26^. Among those selected based on expert opinion, *percent of the female population* is due to the fact that women’s vulnerability and discrimination tend to become more visible during periods of societal disruption, such as catastrophic disasters, when the social structure is disturbed^38^. During and after the disaster, pregnancy, childbirth, insecurity, and more exposure to sexual violence, health, and coping mechanisms after a shock are some factors that make a woman population vulnerable in critical situations^38^. Such a reduction in the data dimension based on expert opinion ensures the avoidance of redundancy and irrelevance in underlying variables^39,40^. These variables will then go through a round of mixing to create 22 mixed variables that combine information at subcategories. For example, we combine the three original subcategories of income less than 10 K, income between 10 K and 20 K, and income between 20 K and 30 K into a mixed variable called low income (Table 1).

**Table 1:** List of selected variables under each category

Demographics	Socio-economic status	Total population	Population per units	Hazus 5.0, which was released on April 30, 2021, is based on 2010 Census data. Modified by National Structure Inventory (NSI) data developed by the U.S. Army Corp of Engineers Hydrologic Engineering Center, Flood Impact Assessment (USACE HEC-FIA) in coordination with FEMA.
		Total units		
		Income less than 10 K	Low-income	
		Income between 10 K and 20 K		
		Income between 20 K and 30 K		
		Income between 75 K and 100 K	High-Income	
		Income Over 100k		
		Renter occupied multi-family units	Renter percent	
		Renter occupied single-family units		
		Owner-occupied multi-family units	Owner percent	
		Owner-occupied single-family units		
		Average cash rent		
		Average home value		
	Minority	Population stating Asian	Minority percent	
		Population stating Black		
		Population stating Hispanic		
		Population stating Native American		
		Population stating Pacific Islander		
		Population stating other races only		
	Household	Population less than 16 years-old percent		
		Population over 65 years-old percent		
		Female population percent		
Infrastructure	General Building Stuck	Units built before 1940	Old build units percent	Hazus 5.0, based on RSMeans (a construction cost estimators toolbox) version 2018.
		Units built between 1940 and 1949		
		Units built between 1950 and 1959		
		Units built between 1980 and 1989	Recent build units percent	
		Units built between 1990 and 1998		
		Units built after 1998		
		Multi-dwellings (10 to 19 units)	Multi_dwelling building count (more than 10 units)	
		Multi-dwellings (20 to 49 units)		
		Multi-dwellings (50+ units)		
		Manufactured housing		
		Churches and other non-profit org.		
		Nursing home building counts		
	Essential Facilities	Distance to the nearest emergency operation centers		Hazus 5.0 has been updated by 2019 Homeland Infrastructure Foundation-Level Data (HIFLD).
		Distance to the nearest medical care		
		Distance to the nearest shelters		
		Distance to the nearest fire stations		
		Distance to the nearest police stations		

*Small hospitals, Medium hospitals, Large hospitals, Medical offices, and Nursing homes are included under Medical care facilities.

Nonurban/Urban Fire Station for Volunteer Fire Departments and for Traditional Fire Departments are included under Fire station facilities.

Nonurban/Urban police stations, state prisons, and county/local jails are included under Police station facilities.

Nonurban/Urban Local EOC, state EOC, FEMA Area Offices, and FEMA Regional and National Offices are included under Emergency Operations Centers.

To prevent the range variability from dominating the process of selection based on their relative contribution to overall vulnerability, we normalize each of the selected/mixed variables through a min–max feature scaling equation as:

1 $${x}^{{\prime} }=\frac{x-{{\min }}\left(x\right)}{{{\max }}\left(x\right)-{{\min }}\left(x\right)}$$

where $x$ is the original value of the mixed variables, and ${x{{\hbox{'}}}}$ is the normalized value. Such normalization helps deal with different measuring units and helps with outlier problems^41,42^. These mixed variables then need to be fed into an ML algorithm (here, random forest regression) to determine the relative contribution of each to the estimated SEIV. To prepare the set of selected variables for regression analysis, we first need to evaluate the severity of multicollinearity among the variables involved. Within a given dataset, highly correlated variables may contain similar information about the variance and thus may adversely affect the efficiency of regression analysis^43,44^. For this purpose, we have implemented the VIF, which helps detect the multicollinearity of variables within a given dataset and avoids collinearity problems in regression^45^. VIF is calculated as:

2 $${{{{{{\rm{VIF}}}}}}}_{{{{{{\rm{i}}}}}}}=\,1/(1-{R}_{i}^{2})$$

where *R*_*i*_ is the coefficient of determination of the regression between each variable and all other predictor variables. Large VIF values (i.e., >10) indicate that there is significant multicollinearity in the dataset that needs to be considered by removing the correlated variables^45^. By implementing VIF, we detected multicollinearity in a set of multiple regression variables. Therefore, we removed the components with VIFs above 10 from the index calculation and left them with 19 final variables.

To determine the contribution of the final selected variables to the SEIV index, we used a random forest (RF) algorithm. RF is an ensemble learning method that uses a multitude of decision trees at training time to improve the performance of the model^31^. RF can be used for both classification and regression problems. The output of RF is the mean prediction of the individual trees for the regression task^46^. The RF algorithm has a built-in feature importance that describes which features are more relevant. The feature selection for internal nodes of each tree is based on variance reduction for the regression task. Thus, for each feature, we can measure how, on average, it decreases the impurity. The highest decrease is the most relevant and important^47^. To calculate the feature importance, we train and validate the RF algorithm using storm property damage data provided via the NOAA Storm Events database. Since the damage estimates are reported at specific latitudes and longitudes, we need to define a buffer zone around the reporting point to match the damages to our vulnerability estimates within that given area. In fact, the buffer zone serves as a spatial sampling domain over which blocks carry vulnerability information associated with the reported damage. We found a buffer zone with a radius of 0.1 degrees around the reporting point suitable for selecting blocks that could have significantly contributed to the reported damage during an event.

For a reported damaging event at a given location, the estimated damage and vulnerability may not be identical, and hazard characteristics play a major role in the extent of damage observed. Thus, we have generated 1000 synthetic hazard scenarios and picked the ensemble median of resulting weights for vulnerability calculation. In the absence of real hazard intensity observation and a unifying system that can make comparable hazard information amongst various types of hazards (i.e., tornados, wildfire, and floods), these synthetic hazard scenarios probabilistically estimate the range of possibilities for the underlying hazard intensities that could have contributed to this given level of damage. To represent the interconnectedness between hazard and damage in the process of hazard scenario generation, we follow this recipe for generating synthetic scenarios using generalized extreme value (GEV) distribution. There are several studies that support the application of GEV to describe the underlying distribution of different hazards (see KJ Beven et al. 2018, for example). To determine the location parameter of GEV, we multiply the damage values by a fixed number (here 0.33) to ensure a strong correlation exists between damage and the synthetic hazard; then normalize it for statistical analysis. Next, for a given location, we generate 1000 random hazards with scale parameters ranging between 0.05 and 0.1. Hazard scenarios generated by GEV with scale parameters beyond this range no longer seem to be related to damage, so considering them in ML would not be meaningful. Those generated synthetic hazard scenarios are going to be considered as an input feature to the RF algorithm. The ensemble median of 1000 simulations would provide the importance weights for SEIV index calculations.

Using a trial and error approach, we consider 80 decision trees for the algorithm, 80% of the data for training, and 20% for testing the model. To evaluate the performance of the algorithm, we used K-fold cross-validation (with K = 10). K-fold cross-validation is a method commonly used to estimate the skill of ML models on unseen data. The data set is first randomly divided into k subsets with equal sample sizes. Then, each fold is taken as a test data set, and the other k-1 fold is taken as a training set. The model is fit to the training data and evaluated on the test data^48^. The performance of the model is the mean of the model skill scores of each k-fold cross-validation run. This approach helps stabilize the variability of accuracy estimates^33^. Then, the estimated weight from the RF algorithm (Wi) for each select variable i will be used to calculate the SEIV index for each block in CONUS by:

3 $${{{{{\rm{SEIV}}}}}}=\mathop{\sum }\limits_{i=1}^{i=19}\left({W}_{i}{X^{\prime} }_{i}\right)$$

where ${{X{{\hbox{'}}}}}_{i}$ is the normalized magnitude of the selected variable ${X}_{i}$. SEIV ranges from 0 to 1; the higher the number is, the more vulnerable the block is estimated to be against natural hazards.

In summary, the SEIV index is obtained using the following steps: (1) Normalize each of the selected/mixed variables through min–max feature scaling to prevent the range variability of components. $({X^{\prime} }_{i})$, (2) We implement the VIF to evaluate the severity of multicollinearity among the variables and then remove the highly correlated variables, (3) the RF algorithm was performed to determine the contribution of the final selected variables $({W}_{i})$, (4) Interpret the influence of each component on socioeconomic-infrastructure vulnerability and assign signs of positive/negative contributions to them, (5) calculate the SEIV index for each block in the CONUS as the sum of multiplying the estimated weight from the RF algorithm $({W}_{i})$ for every 19 variables by their normalized variables $({X^{\prime} }_{i})$.

The blocks around the CONUS are classified based on their estimated ${{{{{\rm{SEIV}}}}}}$ quantile to very low (${{{{{{\rm{q}}}}}}}_{{{{{{\rm{SEIV}}}}}}}$ < 0.2), low (0.2 < $\,{{{{{{\rm{q}}}}}}}_{{{{{{\rm{SEIV}}}}}}}$ < 0.4), medium (0.4 < $\,{{{{{{\rm{q}}}}}}}_{{{{{{\rm{SEIV}}}}}}}$ < 0.6), high (0.6 < $\,{{{{{{\rm{q}}}}}}}_{{{{{{\rm{SEIV}}}}}}}$ < 0.8), and very high (0.8 < $\,{{{{{{\rm{q}}}}}}}_{{{{{{\rm{SEIV}}}}}}}$).

### Reporting summary

Further information on research design is available in the Nature Portfolio Reporting Summary linked to this article.

## Supplementary information

Reporting Summary

## Data Availability

All the data used in this study are publicly accessible. The latest version of Hazus data and software is publicly accessible by FEMA Flood Map Service Center via the following link: https://www.fema.gov/flood-maps/tools-resources/flood-map-products/hazus/software. The Comprehensive Data Management System (CDMS) software provided by “informer technologies Inc” that is available via the following link: https://comprehensive-data-management-system-cdm.software.informer.com/ is helpful for downloading and managing Hazus data. The census shape file data, provided by U.S Census Bureau, is available via the following link: https://www.census.gov/geographies/mapping-files/time-series/geo/tiger-line-file.html. The NOAA storm event database is publicly accessible via NOAA National Centers for Environmental Information website at this link: https://www.ncdc.noaa.gov/stormevents/. Due to the exceptionally large size of the data set produced in this study, the complete vulnerability data cannot be openly shared. However, the vulnerability of New York State as a selected region is available through the figshare data repository: 10.6084/m9.figshare.22647937.
