# Supplementary material for: Block-level vulnerability assessment reveals disproportionate impacts of natural hazards across the conterminous United States
Source: Nat Commun. 2023 Jul 14;14:4222. doi: 10.1038/s41467-023-39853-z (PMC10349093; doi:10.1038/s41467-023-39853-z)
Supplement: Supplementary file 1 — Reporting Summary [file 41467_2023_39853_MOESM1_ESM.pdf]

nature portfolio

Corresponding author(s): Hamid Moradkhani

Last updated by author(s): Apr 13, 2023

Reporting Summary

Nature Portfolio wishes to improve the reproducibility of the work that we publish. This form provides structure for consistency and transparency in reporting. For further information on Nature Portfolio policies, see our Editorial Policies and the Editorial Policy Checklist.

Statistics

For all statistical analyses, confirm that the following items are present in the figure legend, table legend, main text, or Methods section.

n/a	Confirmed	
☐	☒	The exact sample size (*n*) for each experimental group/condition, given as a discrete number and unit of measurement
☒	☐	A statement on whether measurements were taken from distinct samples or whether the same sample was measured repeatedly
☒	☐	The statistical test(s) used AND whether they are one- or two-sided<br>*Only common tests should be described solely by name; describe more complex techniques in the Methods section.*
☒	☐	A description of all covariates tested
☒	☐	A description of any assumptions or corrections, such as tests of normality and adjustment for multiple comparisons
☐	☒	A full description of the statistical parameters including central tendency (e.g. means) or other basic estimates (e.g. regression coefficient) AND variation (e.g. standard deviation) or associated estimates of uncertainty (e.g. confidence intervals)
☒	☐	For null hypothesis testing, the test statistic (e.g. *F*, *t*, *r*) with confidence intervals, effect sizes, degrees of freedom and *P* value noted<br>*Give P values as exact values whenever suitable.*
☒	☐	For Bayesian analysis, information on the choice of priors and Markov chain Monte Carlo settings
☒	☐	For hierarchical and complex designs, identification of the appropriate level for tests and full reporting of outcomes
☒	☐	Estimates of effect sizes (e.g. Cohen's *d*, Pearson's *r*), indicating how they were calculated

Our web collection on statistics for biologists contains articles on many of the points above.

Software and code

Policy information about availability of computer code

Data collection	- We have used the data available in Hazus 5.0, released on April 30, 2021.<br>- To search for and transfer data out of a given Hazus state inventory dataset, we have used Comprehensive Data Management System (CDMS) tool, version v3.0.<br>- We have, also, used census data, available via U.S Census Bureau (2010).
Data analysis	- We have used Python package "sklearn.ensemble.RandomForestRegressor" via "scikit-learn 1.2.2" . The package is available via the following link;<br>https://scikit-learn.org/stable/modules/generated/sklearn.ensemble.RandomForestRegressor.html#sklearn-ensemble-randomforestregressor<br>- We have, also, used the package "numpy.random.gumbel" via "NumPy v1.24" for GEV random hazard scenarios. The package is available via the following link:<br>https://numpy.org/doc/stable/reference/random/generated/numpy.random.gumbel.html

For manuscripts utilizing custom algorithms or software that are central to the research but not yet described in published literature, software must be made available to editors and reviewers. We strongly encourage code deposition in a community repository (e.g. GitHub). See the Nature Portfolio guidelines for submitting code & software for further information.

Data

Policy information about availability of data

All manuscripts must include a data availability statement. This statement should provide the following information, where applicable:

- Accession codes, unique identifiers, or web links for publicly available datasets
- A description of any restrictions on data availability
- For clinical datasets or third party data, please ensure that the statement adheres to our policy

All the data used in this study are publicly available via following sources;

- Users can download the latest version of Hazus software for free from "FEMA Flood Map Service Center" via the following link; https://www.fema.gov/flood-maps/tools-resources/flood-map-products/hazus/software
- Users can download the latest version of Comprehensive Data Management System (CDMS) software from "informer technologies,Inc" via the following link; https://comprehensive-data-management-system-cdm.software.informer.com/
- Users can download census shape file data, available via U.S Census Bureau, through the following link; https://www.census.gov/geographies/mapping-files/time-series/geo/tiger-line-file.html
- Users can access NOAA storm event database from "NOAA National Centers for Environmental Information" website. https://www.ncdc.noaa.gov/stormevents/

Human research participants

Policy information about studies involving human research participants and Sex and Gender in Research.

Reporting on sex and gender	N/A
Population characteristics	N/A
Recruitment	N/A
Ethics oversight	N/A

Note that full information on the approval of the study protocol must also be provided in the manuscript.

Field-specific reporting

Please select the one below that is the best fit for your research. If you are not sure, read the appropriate sections before making your selection.

☐ Life sciences ☒ Behavioural & social sciences ☐ Ecological, evolutionary & environmental sciences

For a reference copy of the document with all sections, see nature.com/documents/nr-reporting-summary-flat.pdf

Behavioural & social sciences study design

All studies must disclose on these points even when the disclosure is negative.

Study description	It is quantitative analysis of Socio-Economic_Infrustructural Vulnerability.
Research sample	The descriptive social and economic characteristics data at the census block level were obtained from Hazus data inventaory. The data available in Hazus 5.0, released on April 30, 2021, contain 59 and 33 demographic and building count variables, respectively, for 10,946,768 blocks over the CONUS. It is based on 2010 census data and modified by the National Structure Inventory (NSI) data developed by the United States Army Corp of Engineers Hydrologic Engineering Center, Flood Impact Assessment (USACE HEC-FIA).
Sampling strategy	With almost 11 million blocks involved no minimum sample size analysis needed. For calibration /validation we use the National Oceanic and Atmospheric Administration (NOAA) Storm Events Database, a comprehensive repository that provides information for different types of natural disasters, between 2006 and 2020 with ~130,000 events that meet our criteria for inclusion (estimated damage between $10,000 and $250 million.
Data collection	We obtained descriptive components (demographics and building count by census block) from the Hazus data inventory through the Comprehensive Data Management System (CDMS). The data available in Hazus 5.0, released on April 30, 2021, contain 59 and 33 demographic and building count variables, respectively, for 10,946,768 blocks over the CONUS. It is based on 2010 census data and modified by the National Structure Inventory (NSI) data developed by the United States Army Corp of Engineers Hydrologic Engineering Center, Flood Impact Assessment (USACE HEC-FIA).

Timing	We have not collected the data; the data are publicly available. the demographic information is based on census 2010 and damage reports are from 2006 to 2020.
Data exclusions	From the NOAA storm event databased events with estimated damage less than $10,000 or greater $250 million were excluded to ensure the regression analysis results remained valid over the range of common possibilities.
Non-participation	There is no participants were involved in the study.
Randomization	Our data are publicly available, and we have not collected the data by ourselves, so the randomization was not applied to this study.

Reporting for specific materials, systems and methods

We require information from authors about some types of materials, experimental systems and methods used in many studies. Here, indicate whether each material, system or method listed is relevant to your study. If you are not sure if a list item applies to your research, read the appropriate section before selecting a response.

Materials & experimental systems

n/a	Involved in the study
☒	☐ Antibodies
☒	☐ Eukaryotic cell lines
☒	☐ Palaeontology and archaeology
☒	☐ Animals and other organisms
☒	☐ Clinical data
☒	☐ Dual use research of concern

Methods

n/a	Involved in the study
☒	☐ ChIP-seq
☒	☐ Flow cytometry
☒	☐ MRI-based neuroimaging
